# NIHR surgery trials

**DOI:** 10.1308/003588413X13511609955698

**Published:** 2013-01

## Abstract

The *Annals* exists in part to keep surgeons up to date with advances in surgical research. We also have a responsibility to inform our readers of research opportunities that they may wish to join. Professor John Scholefield heads the National Institute for Health Research (NIHR) National Surgery Specialty Group and he has asked us to publish regular updates of surgical research activity taking place across the UK. We are pleased to do so and provide some additional information below.

The Comprehensive Clinical Research Network (CCRN) is one of eight NIHR networks in England that provide researchers with the practical support they need to make clinical trials happen in the NHS so that more patients can take part in clinical research in England. The devolved nations have similar arrangements in place to support clinical research.

The Surgery Specialty Group is 1 of 24 specialty groups that provide national networks of topic-specific expertise. These groups are key to the success of the CCRN. They work at both national and local levels to ensure the successful delivery of research in their specialties. This means ensuring that studies are delivered to target and on time.

> We offer opportunities for surgeons and other healthcare professionals to become involved in research as well as for existing researchers to access a network of skilled research support staff and wider patient populations.
> Comprehensive Local Research Networks can provide: assistance with the research and development approvals process; research nurses to consent patients into studies and perform study-related procedures; support for services such as pharmacy, imaging and pathology; and access to training opportunities.
> Detailed information about specialty groups including contact details for regional representatives can be found on the Clinical Research Network website (http://www.crncc.nihr.ac.uk/about_us/ccrn/specialty/) or you can email the CCRN specialty groups team (crncc.specialty@nihr.ac.uk).

The Surgery Specialty Group distributes a list of trials led by surgeons that are open to recruitment to raise awareness among practising surgeons. This list will now be published in the *Annals* on a regular basis. We hope this will aid recruitment to surgical trials and provide opportunities for surgeons to get involved in research.

**Table 1:** Surgery portfolio: studies open to new sites

Portfolio ID	Project	Contact	Open to new sites?
8299	eTHoS: Comparing stapled haemorrhoidopexy with traditional excisional surgery for haemorrhoidal disease	Hanne Bruhn h.bruhn@abdn.ac.uk	Yes, within and outside lead country
9592	FIAT: Fistula-in-ano trial comparing Surgisis^®^ anal fistula plug versus surgeon’s preference for transsphincteric fistula-in-ano	Laura Magill e.l.magill@bham.ac.uk	Yes, within lead country only
11783	WOLLF: Standard wound management versus negative pressure wound therapy in the treatment of adult patients with an open fracture of the lower limb	Matthew Costa matthew.costa@warwick.ac.uk	Yes, within lead country only
11911	BY-BAND: Gastric bypass or adjustable gastric banding surgery to treat morbid obesity	Jane Blazeby j.m.blazeby@bristol.ac.uk	Yes, within lead country only
12486	HubBLe: Comparing rubber band ligation with haemorrhoidal artery ligation in the management of symptomatic second and third degree haemorrhoids	Katie Biggs c.e.biggs@sheffield.ac.uk	Yes, within lead country only

The lead country is the home nation (England, Wales, Scotland or Northern Ireland) of the Chief Investigator.

For further details:

> Get in touch with the named contact
> Click on the link for each trial (if viewing online)
> Visit the website of the Clinical Research Network Study Portfolio (http://public.ukcrn.org.uk/search/)

